# Current challenges in photosynthesis: from natural to artificial

**DOI:** 10.3389/fpls.2014.00232

**Published:** 2014-05-28

**Authors:** Harvey J. M. Hou, Suleyman I. Allakhverdiev, Mohammad M. Najafpour, Govindjee

**Affiliations:** ^1^Department of Physical Sciences, Alabama State UniversityAlabama, AL, USA; ^2^Institute of Plant Physiology, Russian Academy of SciencesMoscow, Russia; ^3^Institute of Basic Biological Problems, Russian Academy of SciencesMoscow, Russia; ^4^Department of Chemistry, Center of Climate Change and Global Warming, Institute for Advanced Studies in Basic SciencesZanjan, Iran; ^5^Departments of Biochemistry and Plant Biology, Center of Biophysics and Quantitative Biology, University of Illinois at Urbana-ChampaignUrbana, IL, USA

**Keywords:** photosynthesis, artificial photosynthesis, water oxidation, thylakoid, chlorophyll *f*

Photosynthesis is a process by which plants, algae, cyanobacteria, and anoxygenic photosynthetic bacteria capture and store solar energy on a massive scale, in particular via the water-splitting chemistry (Hoganson and Babcock, 1997; Blankenship, 2002; Ferreira et al., 2004; Loll et al., 2005; Yano et al., 2006; Umena et al., 2011). It is the most important reaction on Earth, estimated to produce more than 100 billion tons of dry biomass annually; this means that photosynthesis is producing biomass equal to two Egyptian pyramids per hour. But, this will not be enough to sustain life on Earth by the year 2050. The global fossil fuels on which we currently depend are derived from millions of years of past photosynthetic activity. The fossil energy fuels are limited and must be replaced by renewable and environment-friendly energy source to support and sustain life on Earth (Lewis and Nocera, 2006; Blankenship et al., 2011). To address this immediate energy crisis, worldwide efforts are being made on artificial photosynthesis using the principles and mechanisms observed in nature (Brimblecombe et al., 2009; McConnell et al., 2010; Kanady et al., 2011; Wiechen et al., 2012; Najafpour et al., 2013). It is not a matter of mimicking natural photosynthesis, but to use its current knowledge to improve photosynthesis itself, as well as to produce biofuels, including hydrogen evolution by artificial means (Barber, 2009; Hou, 2010; Nocera, 2012; He et al., 2013).

This book contains 10 chapters and presents recent advances in photosynthesis and artificial photosynthesis. It starts with two opinion articles on possible strategies to improve photosynthesis in plants and fascinating mechanisms of unidirectional photodamage of pheophytin in photosynthesis. The idea that plant photosynthesis is maximized due to the perfect evolution might be faulty. Leister evaluated and argued the issue openly and proposed that improvement of photosynthesis can be made by synthetic biology including genetic engineering, redesign or de novo creation of entire photosystems as well as conventional breeding (Leister, 2012). Unidirectional photodamage of a pheophytin molecule in photosystem II and purple bacterial reaction centers was observed. The mysterious phenomena were analyzed and discussed in terms of different possible functions of the pheophytin in photosynthesis (Hou, 2014).

The book is followed by four review articles that discuss the current state of research on: photosynthetic water oxidation in natural and artificial photosynthesis, as obtained by mass spectrometry (MS) and Fourier transform infrared spectroscopy (FTIR); functional models of thylakoid lumen; and horizontal gene transfer in photosynthetic eukaryotes. The time-resolved isotope-ratio membrane-inlet mass spectrometry (TR-IR-MIMS) is able to determine the isotopic composition of gaseous products. Shevela et al briefly introduced the key aspects of the methodology, summarized the recent results on the mechanisms and pathways of oxygen formation in PS II using this unique technique and outlined the future perspectives of the application in water splitting chemistry (Shevela and Messinger, 2013) Another unique technique in probing the mechanism of water oxidation in PS II is the light-induced FTIR difference spectroscopy. Chu reviewed the recent fruitful structural data, and believed that the FTIR will continue to provide vital structural and mechanistic insights into the water-splitting process in PS II together with isotopic labeling, site-directed mutagenesis, model compound studies, and computational calculation (Chu, 2013). The thylakoid lumen offers the environment for oxygen evolution, electron transfer, and photoprotection in photosynthesis. Jarvi et al evaluated the recent studies of many lumen proteins and highlighted the importance of the thiol-disulfide modulation in controlling the functions of the thylakoid lumen proteins and their pathways of photosynthesis (Järvi et al., 2013). Qiu et al discussed that importance of the horizontal gene transfer (HGT) in enriching the algal genomes and proposed that the alga endosymbionts may be the HGT vectors in photosynthetic eukaryotes (Qiu et al., 2013).

Finally, the book offers four research articles, which focus on FTIR studies on photosynthetic reaction centers, functions of thylakoid protein kinases STN7 and STN8, photosynthesis acclimation of maize seedlings, and characterization of the newly discovered chlorophyll *f*-containing cyanobacterium *Halomicronema hongdechloris*. The computational calculation (ONIOM) is increasingly critical in interpreting the FTIR data in elucidating the structural and functional relationship in photosynthesis. Zhao et al using ONIOM type calculation to simulate isotope edited FTIR difference spectra for reaction centers with a variety of foreign quinones in the Q_A_ site and allows a direct assessment of the appropriateness of previous IR assignments and suggestions (Zhao et al., 2013). The protein kinases STN7 and STN8 are predominately responsible for the thylakoid phosphorylation in PS II. Wunder et al reported the effects of the STN8 expression levels on the formation and modulation of thylakoid proteins and kinases (Wunder et al., 2013). Hirth et al assessed the photosynthetic acclimation responses of the C3 and C4 plants under simulated field light conditions (Hirth et al., 2013). Recently a chlorophyll *f* (Chl *f*) in cyanobacterium *Halomincronema hongdechloris* was identified and has the most red-shifted absorption peak of 707 nm in oxygenic photosynthesis (Chen et al., 2010), which may enhance the potential photosynthesis efficiency for solar fuel production. The *Halomincronema hongdechloris* was characterized upon the exposure to the different light, pH, salinity, temperature, and nutrition to achieve the optimizing growth culture conditions (Li et al., 2014).

Due to the extremely limited time frame for collecting manuscripts and the strict deadline for publishing this book, several planned manuscripts by world leaders, who had agreed to contribute, are unfortunately not included in this book. Thus, the current book provides a snapshot of the latest work in photosynthesis research. To obtain complete information on the current progress in the field of photosynthesis, we highly recommend reviews and research articles, published in 2013, in two volumes of *Photosynthesis Research* (Allakhverdiev et al., 2013a,b)

In conclusion, the book provides readers with some of the most recent and exciting breakthroughs from natural to artificial photosynthesis, discusses the potential limitations of the results, and addresses open questions in photosynthesis and energy research. It is written by 31 young active scientists and established leading experts from Australia, Finland, Germany, Sweden, Taiwan, and the United States. We hope that this book is able to provide novel and insightful information to readers and stimulate the future research endeavors in the photosynthesis community.

## Conflict of interest statement

The authors declare that the research was conducted in the absence of any commercial or financial relationships that could be construed as a potential conflict of interest.

## References

[B1] Allakhverdiev S. I. Shen J.-R. Edwards G. E. (2013a). Special issues on photosynthesis education honoring govindjee. Photosynthesis Res. 116, 1–534 23990372 10.1007/s11120-013-9913-3

[B2] Allakhverdiev S. I. Shen J.-R. Edwards G. E. (2013b). Special issues on photosynthesis education honoring govindjee. Photosynthesis Res. 117, 1–566 23990372 10.1007/s11120-013-9913-3

[B3] Barber J. (2009). Photosynthetic energy conversion: natural and artificial. Chem. Soc. Rev. 38, 185–196 10.1039/b802262n 19088973

[B4] Blankenship R. E. (2002). Molecular Mechanisms of Photosynthesis. Oxford: Blackwell Science 10.1002/9780470758472

[B5] Blankenship R. E. Tiede D. M. Barber J. Brudvig G. W. Fleming G. Ghirardi M. (2011). Comparing the efficiency of photosynthesis with photovoltaic devices and recognizing opportunities for improvement. Science 332, 805–809 10.1126/science.1200165 21566184

[B6] Brimblecombe R. Dismukes G. C. Swiegers G. F. Spiccia L. (2009). Molecular water-oxidation catalysts for photoelectrochemical cells. Dalton Trans. 43, 9374–9384 10.1039/b912669d 19859588

[B7] Chen M. Schliep M. Willows R. D. Cai Z. L. Neilan B. A. Scheer H. (2010). A red-shifted chlorophyll. Science 329, 1318–1319 e.1 10.1126/science.1191127 20724585

[B8] Chu H.-A. (2013). Fourier transform infrared difference spectroscopy for studying the molecular mechanism of photosynthetic water oxidation. Front. Plant Sci. 4:146 10.3389/fpls.2013.00146 23734156 PMC3659307

[B9] Ferreira K. N. Iverson T. M. Maghlaoui K. Barber J. Iwata S. (2004). Architecture of the photosynthetic oxygen-evolving center. Science 303, 1831–1838 10.1126/science.1093087 14764885

[B10] He W. Zhao K.-H. Hou H. J. M. (2013). Toward solar fuel production using manganese/semiconductor systems to mimic photosynthesis. NanoPhotoBioSciences 1, 63–78

[B11] Hirth M. Dietzel L. Steiner S. Ludwig R. Weidenbach H. Pfalz J. (2013). Photosynthetic acclimation responses of maize seedlings grown under artificial laboratory light gradients mimicking natural canopy conditions. Front. Plant Sci. 4:334 10.3389/fpls.2013.00334 24062753 PMC3770919

[B12] Hoganson C. W. Babcock G. T. (1997). A metallo radical mechanism for the generation of oxygen from water in photosynthesis. Science 277, 1953–1956 10.1126/science.277.5334.1953 9302282

[B13] Hou H. J. M. (2010). Structural and mechanistic aspects of Mn-oxo and Co-based compounds in water oxidation catalysis and potential application in solar fuel production. J. Integr. Plant Biol. 52, 704–711 10.1111/j.1744-7909.2010.00974.x 20666926

[B14] Hou H. J. M. (2014). Unidirectional photodamage of pheophytin in photosynthesis. Front. Plant Sci. 4:554 10.3389/fpls.2013.00554 24454319 PMC3888939

[B15] Järvi S. Gollan P. J. Aro E.-M. (2013). Understanding the roles of the thylakoid lumen in photosynthesis regulation. Front. Plant Sci. 4:434 10.3389/fpls.2013.00434 24198822 PMC3813922

[B16] Kanady J. S. Tsui E. Y. Day M. W. Agapie T. (2011). A synthetic model of the Mn_3_Ca subsite of the oxygen-evolving complex in photosystem II. Science 333, 733–736 10.1126/science.1206036 21817047

[B17] Leister D. (2012). How can the light reactions of photosynthesis be improved in plants? Front. Plant Sci. 3:199 10.3389/fpls.2012.00199 22973282 PMC3428562

[B18] Lewis N. S. Nocera D. G. (2006). Powering the planet: chemical challenges in solar energy utilization. Proc. Natl. Acad. Sci. U.S.A. 103, 15729–15735 10.1073/pnas.0603395103 17043226 PMC1635072

[B19] Li Y. Lin Y. Loughlin P. C. Chen M. (2014). Optimization and effects of different culture conditions on growth of *Halomicronema hongdechloris* – a filamentous cyanobacterium containing chlorophyll *f*. Front. Plant Sci. 5:67 10.3389/fpls.2014.00067 24616731 PMC3934312

[B20] Loll B. Kern J. Saenger W. Zouni A. Biesiadka J. (2005). Towards complete cofactor arrangement in the 3.0A resolution structure of photosystem II. Nature 438, 1040–1044 10.1038/nature04224 16355230

[B21] McConnell I. Li G. Brudvig G. W. (2010). Energy conversion in natural and artificial photosynthesis. Chem. Biol. 17, 434–447 10.1016/j.chembiol.2010.05.005 20534342 PMC2891097

[B22] Najafpour M. M. Mahnaz Abasi M. Allakhverdiev S. I. (2013). Recent proposed mechanisms for biological water oxidation. NanoPhotoBioSciences 1, 79–92

[B23] Nocera D. G. (2012). The artificial leaf. Acc. Chem. Res. 45, 767–776 10.1021/ar2003013 22475039

[B24] Qiu H. Yoon H. S. Bhattacharya D. (2013). Alga lendosymbionts as vectors of horizontal gene transfer in photosynthetic eukaryotes. Front. Plant Sci. 4:366 10.3389/fpls.2013.00366 24065973 PMC3777023

[B25] Shevela D. Messinger J. (2013). Studying the oxidation of water to molecular oxygen in photosynthetic and artificial systems by time-resolved membrane-inlet mass spectrometry. Front. Plant Sci. 4:473 10.3389/fpls.2013.00473 24324477 PMC3840314

[B26] Umena Y. Kawakami K. Shen J. R. Kamiya N. (2011). Crystal structure of oxygen-evolving photosystem II at a resolution of 1.9 A. Nature 473, 55–61 10.1038/nature09913 21499260

[B27] Wiechen M. Berends H.-M. Kurz P. (2012). Water oxidation catalysed by manganese compounds: from complexes to “biomimetic rocks.” Dalton Trans. 41, 21–31 10.1039/c1dt11537e 22068958

[B28] Wunder T. Xu W. Liu Q. Wanner G. Leister D. Pribil M. (2013). The major thylakoid protein kinases STN7 and STN8 revisited: effects of altered STN8 levels and regulatory specificities of the STN kinases. Front. Plant Sci. 4:417 10.3389/fpls.2013.00417 24151498 PMC3801152

[B29] Yano J. Kern J. Sauer K. Latimer M. J. Pushkar Y. Biesiadka J. (2006). Where water is oxidized to dioxygen: structure of the photosynthetic Mn_4_Ca cluster. Science 314, 821–825 10.1126/science.1128186 17082458 PMC3963817

[B30] Zhao N. Lamichhane H. P. Hastings G. (2013). Comparison of calculated and experimental isotope edited FTIR difference spectra for purple bacterial photosynthetic reaction centers with different quinones incorporated into the Q_A_ binding site. Front. Plant Sci. 4:328 10.3389/fpls.2013.00328 24009618 PMC3757576

